# One-step Synthesized Silver Nanoparticles Using Isoimperatorin: Evaluation of Photocatalytic, and Electrochemical Activities

**DOI:** 10.1038/s41598-020-58697-x

**Published:** 2020-02-04

**Authors:** Maryamosadat Mavaei, Azam Chahardoli, Yalda Shokoohinia, Alireza Khoshroo, Ali Fattahi

**Affiliations:** 1 Pharmaceutical Sciences Research Center, Health Institute, Kermanshah University of Medical Sciences, Kermanshah, Iran; 2 Ric Scalzo Botanical Research Institute, Southwest College of Naturopathic Medicine, Tempe, AZ USA; 3 Medical Biology Research Center, Health Technology Institute, Kermanshah University of Medical Sciences, Kermanshah, Iran

**Keywords:** Environmental sciences, Chemistry

## Abstract

In the current study, isoimperatorin, a natural furanocoumarin, is used as a reducing reagent to synthesize isoimperatorin mediated silver nanoparticles (Iso-AgNPs), and photocatalytic and electrocatalytic activities of Iso-AgNPs are evaluated. Iso-AgNPs consisted of spherically shaped particles with a size range of 79–200 nm and showed catalytic activity for the degradation (in high yields) of New Fuchsine (NF), Methylene Blue (MB), Erythrosine B (ER) and 4-chlorophenol (4-CP) under sunlight irradiation. Based on obtained results, Iso-AgNPs exhibited 96.5%, 96.0%, 92%, and 95% degradation rates for MB, NF, ER, and 4-CP, respectively. The electrochemical performance showed that the as-prepared Iso-AgNPs exhibited excellent electrocatalytic activity toward hydrogen peroxide (H_2_O_2_) reduction. It is worth noticing that the Iso-AgNPs were used as electrode materials without any binder. The sensor-based on binder-free Iso-AgNPs showed linearity from 0.1 µM to 4 mM with a detection limit of 0.036 μM for H_2_O_2_. This binder-free and straightforward strategy for electrode preparation by silver nanoparticles may provide an alternative technique for the development of other nanomaterials based on isoimperatorin under green conditions. Altogether, the application of isoimpratorin in the synthesis of nano-metallic electro and photocatalysts, especially silver nanoparticles, is a simple, cost-effective and efficient approach.

## Introduction

Recently, isolated and purified natural products have gained considerable attention in the synthesis and cap of metallic nanoparticles. These natural product-induced metallic nanoparticles (NPIMNPs) have been widely used in the biological, environmental, electrochemical, and catalytic fields^1–8^. Albeit plant extracts are considered as general sources for the green synthesis of metallic nanoparticles^9^, the extracts compositions and concentrations of substances are highly variable, affected by the method of extraction, harvesting season and geographical parameters. As an alternative, the pure natural products with a well–defined concentration of reactant can synthesize reproducible metallic nanoparticles, and the purification of nanoparticles is more facile^10,11^.

Furthermore, physicochemical and biological characteristics of natural products can improve the biological, electrochemical, and catalytic properties of NPIMNPs. In this regard, natural products e.g., phenolic compounds, alkaloids, terpenoids, amino acids, flavonoids, glutathiones, quinones antioxidants, polysaccharides, organic acids, and coumarins have been used^12^. The synthesis of nanoparticles by these natural products is a clean, non-toxic, and environmental-friendly^13^. Compared to their counterparts, synthesized by other methods, NPIMNPs possess improved bioactivities and catalytic characteristics^12^.

Among metallic nanoparticles, silver nanoparticles have been widely studied for catalytic activities. AgNPs can demonstrate light absorption and significant visible light catalytic activity due to its narrow bandgap energy to the many colorant/textile combinations under visible or sunlight illumination^4,14^. The high absorption property of Ag-based NPs in the visible light region, together with preventing the recombination of electron and hole pairs within the photocatalytic process, drew enormous attention in the application of silver nanoparticles for the catalytic field. This aspect of AgNPs makes them an excellent choice for multiple roles in the industrial area^14^. Furthermore, AgNPs have been demonstrated to be good and impressible catalysts for non-enzymatic electrochemical detection of H_2_O_2_^15–20^. A large number of nanostructures decorated with chemically-stabilized AgNPs have been synthesized and used for this porpose^21^.

Current techniques for the synthesis of AgNPs, including photochemical methods, chemical reduction, electron irradiation, gamma irradiation, and laser ablation^22^, are expensive and require high temperature and pressure. Besides, they need toxic and hazardous chemicals like dimethylhydrazine, hydrazine and sodium borohydride and produce toxic organic byproducts that are responsible for various biological risks^23^.

In addition to the synthesis limitations, the stabilization of AgNPs on the electrode surface hampered its application in the field of electrochemistry. In most of the cases, investigators used nonconductive polymers such as Nafion and chitosan as a binder^24–26^. Composites formed with nonconductive binders suffer from their low conductivity, which effects on the electrochemical response of nanocomposite-modified electrode, especially in traces analysis^27,28^. It is expected that the nonconductive materials can decrease the electrochemical performance of the nanomaterials. Therefore, the as-prepared binder-free electrode materials as stable catalysts with high conductivity and active surface area are highly demanded.

To address these unmet technical needs, in the present study, isoimperatorin was applied as a bio-reducing agent; photocatalytic and electrochemical activates of Iso-AgNPs were evaluated. Isoimperatorin is a furanocoumarin isolated from edible Apiaceous plants such as *Angelica dahurica*^29^ and *Prangos ferulacea*^30^. It showed anti-inflammatory, anti-Alzheimer, analgesic, spasmolytic^31,32^, potential cancer prevention^33^, antibacterial and anti-cancer effects^34–37^. There are no reports on photo- or electrochemical-catalytic activities of isoimperatorin. However, there are some reports on electro-catalytic activities of other coumarins as chemo-sensors for the detection of fluoride ions, hydrogen sulfide (H_2_S), mercury (Hg^2+^) ions, hypochlorite, and cyanide anions in aqueous solution^38–42^.

Although the different flavonoids, e.g. hesperidin, naringin, diosmin, quercetin diphosphate, and quercetin pentaphosphate, resveratrol and fisetin have been applied for the synthesis of AgNPs, and catalytic activity of synthesized NPs have been investigated^43,44^, to our best of knowledge, there is no report on the synthesis of silver nanoparticles using isoimperatorin and evaluation of their catalytic properties. Therefore, in this work, we synthesized and characterized Iso-AgNPs using UV–visible spectrophotometer, Fourier-transform infrared spectroscopy (FTIR), scanning electron microscopy (SEM) with energy dispersive X-Ray (EDX) detector X-ray diffraction (XRD), Raman spectroscopy, transmission electron microscopy (TEM) and high-resolution transmission electron microscopy (HRTEM). The electrocatalytic activity of Iso-AgNPs toward H_2_O_2_ reduction and their photocatalytic performance against three toxic mutagenic dye pollutants consist of New-fuchsine (NF), Methylene Blue (MB) and Erythrosine B (ER), and 4-chlorophenol (4-CP) as a non-dye pollutant were evaluated.

## Results and Discussion

### Physical and chemical characterization of Iso-AgNPs

**Figure 1:**
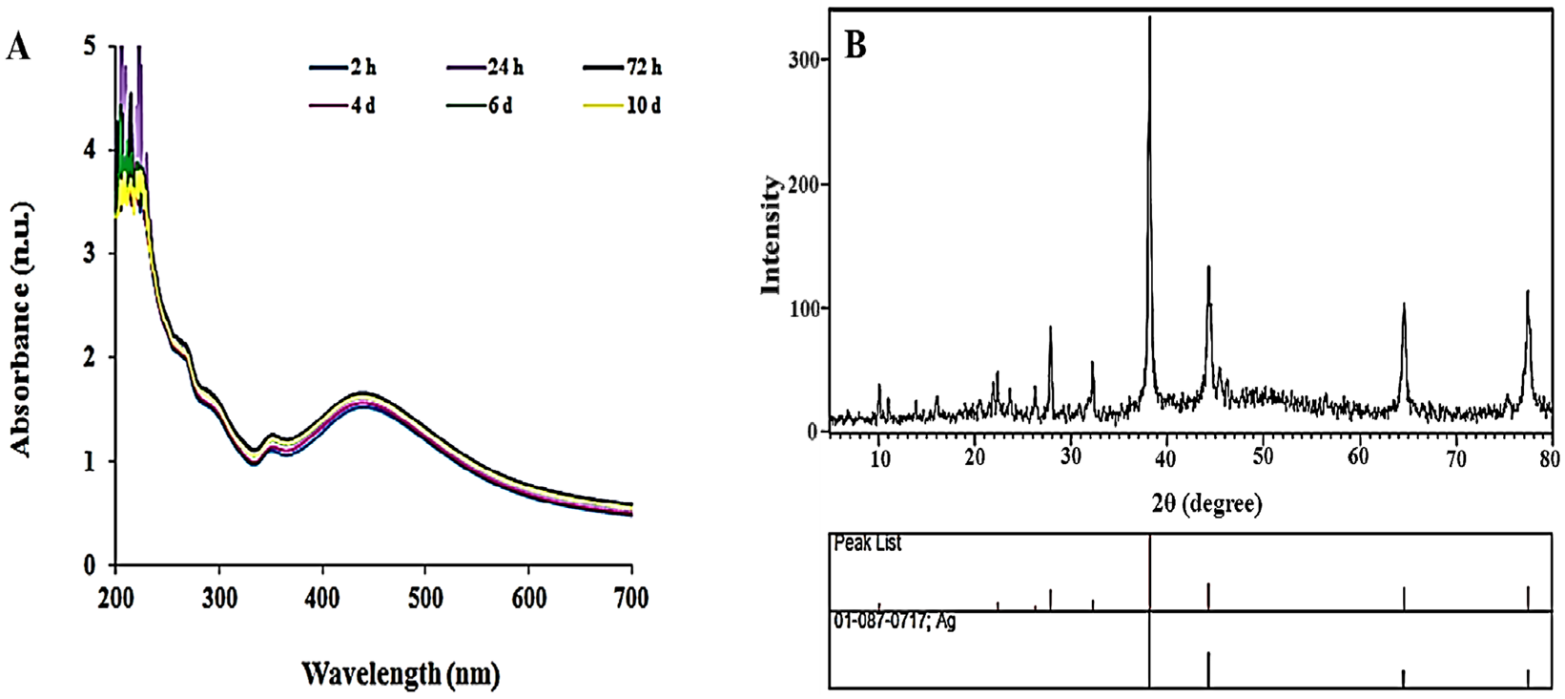
(**A**) UV–visible spectra at different intervals time and (**B**) XRD spectrum of Iso-AgNPs.

The reduction of the Ag^+^ to AgNPs using isoimperatorin was approved by a color change from pale pink to dark brown after two h exposure; the absorption of Iso-AgNPs was shown in Fig. 1. The formation of dark brown color in the reaction mixture, indicating the excitation of Surface Plasmon Resonance (SPR) of Iso-AgNPs is a sign for the synthesis of AgNPs^45,46^. Iso-AgNPs showed a clear and single SPR band with λ_max_ (maximum absorption) at 439 nm (Fig. 1A).

Iso-AgNPs had a face-centered cubic crystalline nature according to the XRD pattern (Fig. 1B). Four reflection peaks were detected in the 2θ range of 20–80°; peaks at 38.1, 44, 64.3, and 77.3° are allocated to the (111), (200), (220), and (311) planes for Iso-AgNPs respectively. The extra picks presented in the spectrum can belong to isoimperatorin^47^.

**Figure 2:**
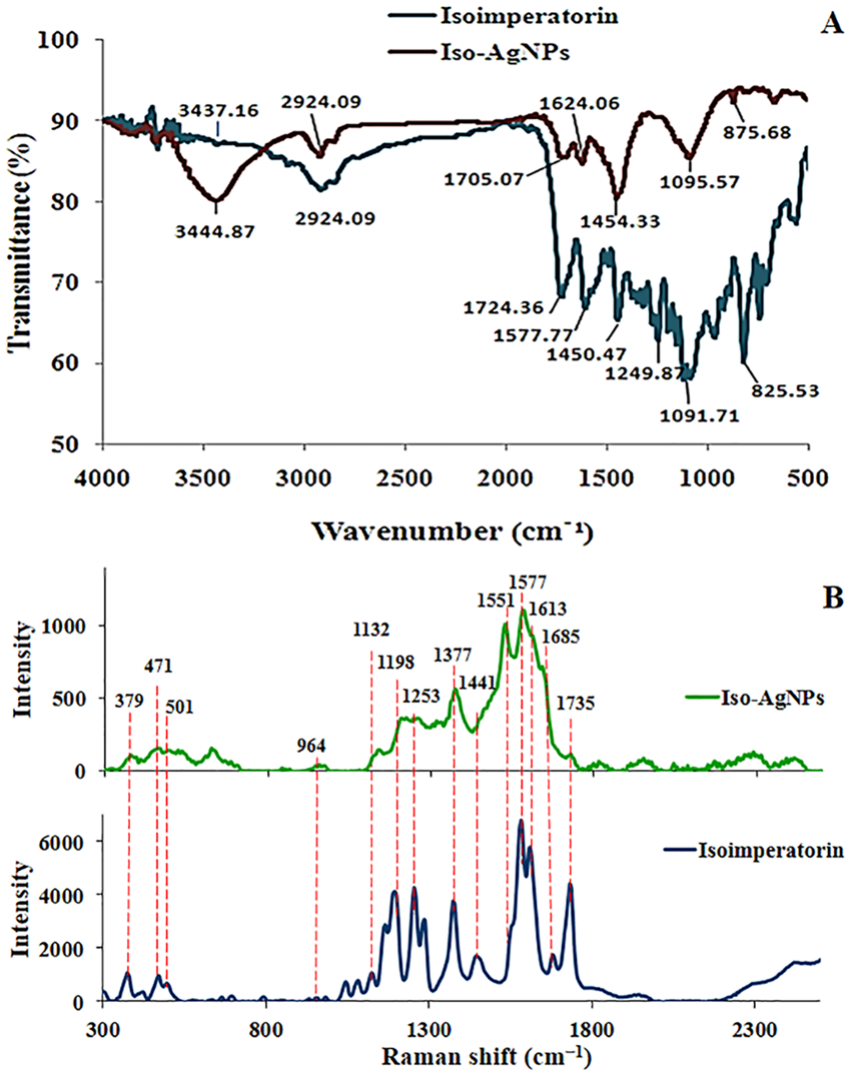
(**A**) FTIR and (**B**) Raman spectra of isoimperatorin and Iso-AgNPs.

**Figure 3:**
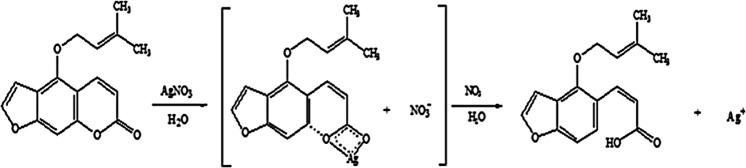
The possible reaction of AgNO_3_ with isoimperatorin.

The possible changes in the structure of isoimperatorin after synthesis of AgNPs were evaluated by FTIR. The FTIR spectra of pure isoimpratorin and synthesized AgNPs are shown in Fig. 2A. Comparing to the isoimperatorin spectrum, some functional groups were disappeared, and new peaks appeared in the FTIR spectrum of Iso-AgNPs. The FTIR spectrum of isoimperatorin indicated the characteristic peak at 1724 cm^−1^ related to stretching vibrations of carbonyl bands from ester group, and the peak at 1249 and 1091 cm^−1^ corresponding to -C-O- of ester group, but the Iso-AgNPs spectrum showed the peak at 1705 cm^−1^ related to stretching vibrations of carbonyl bands from carboxylic acid group, and the peak at 1095 cm^−1^ attributed to C-O- stretching vibration of carboxylic acid group. Also, the broad peak of the OH group at 3444 cm^−1^ was appeared affirming the presence of the carboxylic acid group. Due to the presence of Ag^+^ ions in the stereochemical medium, carboxylate anion is formed, and then carboxylate anion becomes carboxylic acid by the catalytic effect of nitric acid (the byproduct of carboxylate anion). The possible reaction of AgNO_3_ with isoimperatorin is illustrated in Fig. 3. For further characterization of Iso-AgNPs, Raman spectroscopy data has been provided. In Fig. 2B, the Raman spectrum of isoimperatorin, is compared with the spectra of Iso-AgNPs. Characteristic peaks (1685, 1613, 1577, 1551, 1441, 1377, 1253, 1198, 1132, 964, 501, 471, and 379 cm^−1^) from Iso-AgNPs were attributed to isoimperatorin^10,48^^,^. The result indicates that the isoimperatorin is mainly involved in the fabrication of silver nanoparticles. Based on the proposed mechanism for the reaction of AgNO_3_ with isoimperatorin (Fig. 3), the C=O function related to the ester group converts to the carboxylic acid group; therefore, C=O stretching in the Raman spectrum of pure isoimperatorin (1735 cm^−1^) was abolished. These results confirm FTIR results.

**Figure 4:**
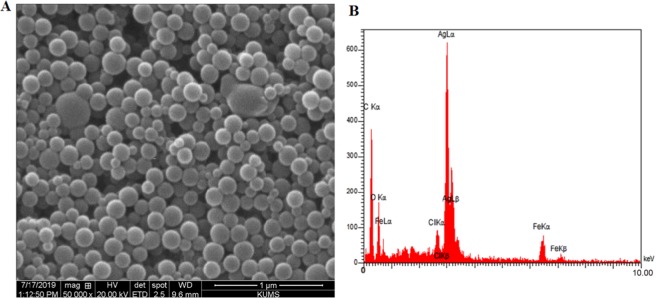
(**A**) SEM micrographs and (**B**) EDX spectra of Iso-AgNPs.

The SEM analysis showed well defined uniformly spherical Iso-AgNPs without any agglomeration and their small size (Fig. 4A). EDX analysis confirmed the presence of elemental silver in the sample (Fig. 4B). The sharp peak at 3.030 keV represented the existence of elemental silver.

**Figure 5:**
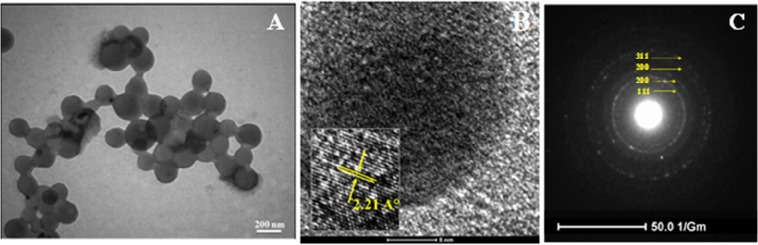
(**A**) TEM, (**B**) HRTEM and (**C**) SAED micrographs of Iso-AgNPs.

The morphology and particle size of Iso-AgNPs were evaluated by TEM image (Fig. 5A); nanoparticles were spherical in shapes. The spherical nanostructure had a diameter range of 79–200 nm. The presence of large particles can be related to slow reaction speed^49,50^. Isoimperatorin solubility in water is low, and using a high concentration of it in the aqueous medium is impossible. Therefore, the rate of nucleation was low, and crystals had enough time for growth.

Figure 5B shows the HRTEM image of the Iso-AgNPs. In inset Fig. 5B, the interplanar distance of 2.21°A can be observed. The crystallinity of Iso-AgNPs was observed by selected area emission diffraction (SAED), which recorded by directing the electron beam perpendicular to NPs. The characteristic fringe array can be indexed as (111), (200), (220) and (311) of the pure face-centered cubic (fcc) lattice structure commonly found for Ag crystal (Fig. 5C). The formation of fcc structured Iso-AgNPs was also confirmed using the XRD patterns.

### Photo-catalytic performance of Iso-AgNPs

**Figure 6:**
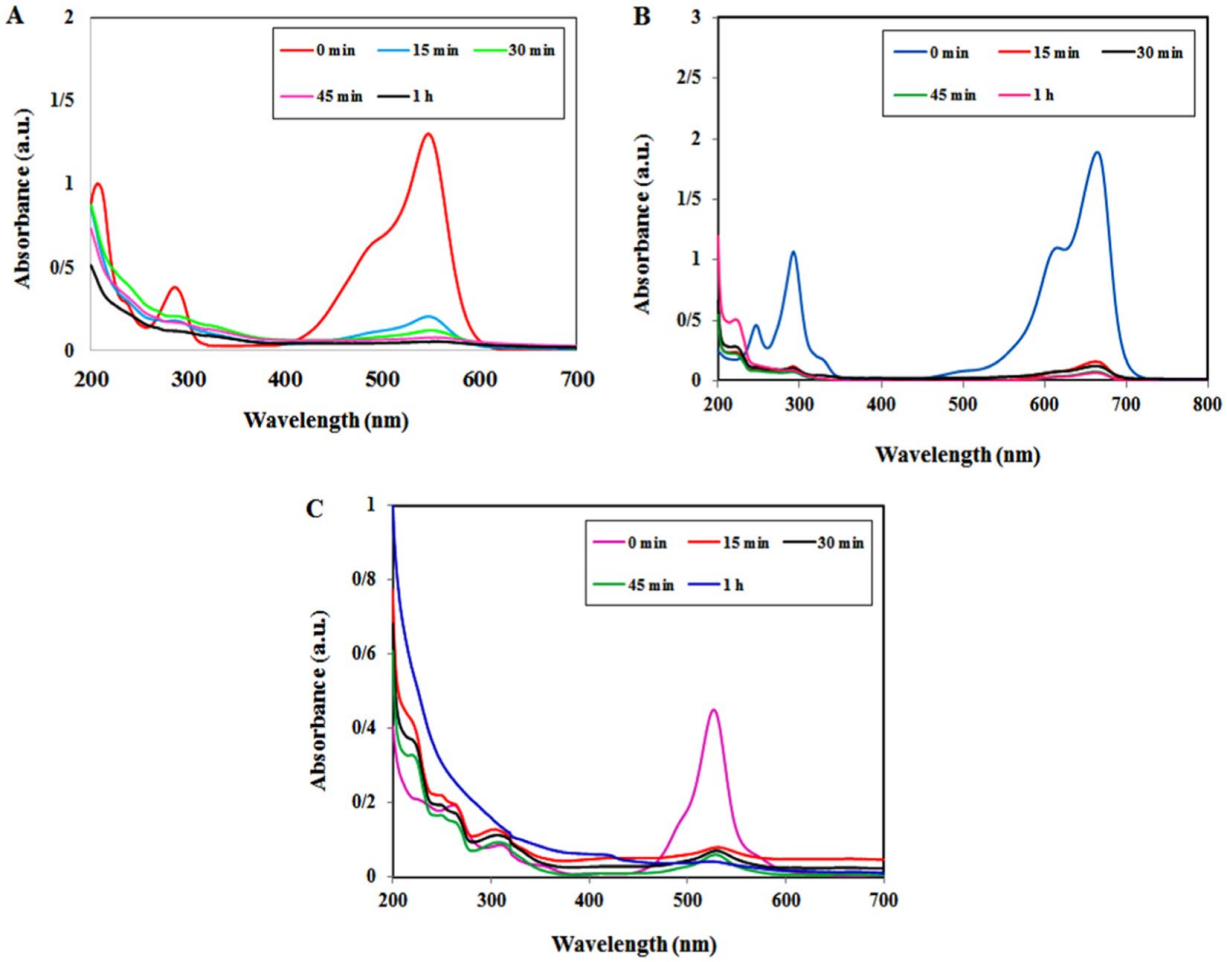
The photocatalytic activity of Iso-AgNPs. UV–vis spectra for photo-degradation of (**A**) NF, (**B**) MB and (**C**) ER Dyes under sun-light irradiation with the Iso-AgNPs.

**Figure 7:**
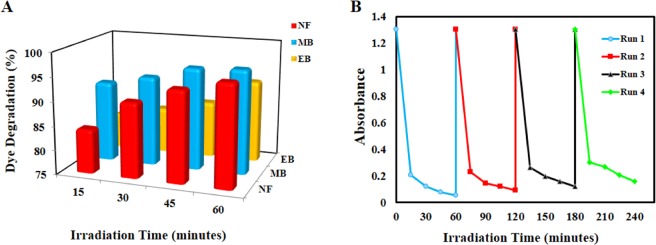
(**A**) The degradation rate (%) of NF, MB and ER dyes over the Iso-AgNPs photocatalyst. (**B**) Reusability of the Iso-AgNPs after four successive runs.

The photocatalytic activity of Iso-AgNPs was analyzed by photo-degradation of NF, MB, ER dyes as a model. As shown in Fig. 6, the degradation of tree dyes under sunlight irradiation initially identified by the color change into light color and then colorless after 60 min for MB, ER, and NF dyes. The UV–vis absorption spectra indicated the decreased peaks for three studied dyes at different time intervals. Initially, the sharp decline occurred for absorption peaks at 548 nm for NF (Fig. 6A), 665 nm for MB (Fig. 6B), and 526 for ER (Fig. 6C) at 15 min under sunlight irradiation. It was continued with the increase of the exposure time. The degradation rates of MB, ER, and NF dyes were 96.5%, 92%, and 96.0%, respectively, at 60 min (Fig. 7A).

In order to examine the reusability and durability of as prepared Iso-AgNPs photocatalyst, a recycling study of Iso-AgNPs was carried out with a stock solution of NF (as a dye molecule model for this section) under identical conditions. Hence, the stability of the Iso-AgNPs was tested for successive four recycling runs. As shown in Fig. 7B, the reusability of the Iso-AgNPs photocatalyst was demonstrated up to a fourth cycle run with a low decrease in photo-degradation efficiency, i.e., 96% to about 88% in the first to fourth cycle runs. The results presented in Fig. 7B demonstrate that the Iso-AgNPs possess robust and excellent cycle stability.

### Possible photo-catalytic mechanism of Iso-AgNPs

**Figure 8:**
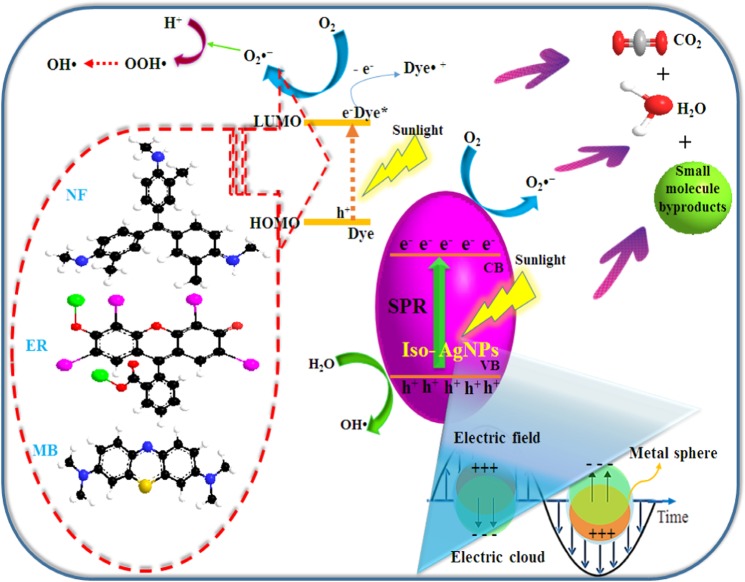
The photo-degradation pathway of dye pollutants in the presence of Iso-AgNPs under sunlight irradiation.

The photocatalytic reaction on the surface of the catalyst generally involves the absorption of light, generation of charge carriers, transport of electron and hole pairs (e^−^_CB_/h^+^_VB_) and surface oxidation-reduction processes. The light from the sunlight irradiation can be absorbed efficiently by both the catalyst and the dye molecules. On irradiation by a photon, Iso-AgNPs absorb the photons with an energy h*ν* and due to their high SPR effect subsequently, generate electrons (e^−^_CB_) and holes (h^+^_VB_) pair (Eq. 1)^51^. The dye molecule can also act as a photosensitizer^52–54^ and it was adsorbed on catalyst surface during the oxidation-reduction experiment (Eqs. 2 and 3). The adsorbed dye molecule (i.e., NF, ER, and MB) was excited by absorbing light and donates its photo-generated electron to the conduction band (CB) of Iso-AgNPs. The photo-generated electrons reduce molecular oxygen (O_2_) adsorbed on the photo-catalyst surface into superoxide radical anions (O_2_^−^•) and hydrogen peroxide radicals (•OOH), quickly (Eqs. 4–7)^53,55^. The photo-generated holes could directly oxidize adsorbed dye molecules or react with surface adsorbed water molecules (H_2_O) or hydroxyl (OH^−^) to generate hydroxyl radicals (HO•) (Eqs. 8, 9 and 10)^55^. As a result, active species (radicals) were generated and assist in the photo-degradation and mineralization of dye molecules (Eq. 11). The possible mechanism for the Iso-AgNPs photodegradation of dyes was proposed in Fig. 8.

1 $${\rm{I}}{\rm{s}}{\rm{o}}-{\rm{A}}{\rm{g}}{\rm{N}}{\rm{P}}{\rm{s}}+{\rm{s}}{\rm{u}}{\rm{n}}{\rm{l}}{\rm{i}}{\rm{g}}{\rm{h}}{\rm{t}}\to {\rm{I}}{\rm{s}}{\rm{o}}-{\rm{A}}{\rm{g}}{\rm{N}}{\rm{P}}{\rm{s}}({{\rm{e}}}_{{\rm{C}}{\rm{B}}}^{-}+{{\rm{h}}}_{{\rm{V}}{\rm{B}}}^{+})$$

2 $${\rm{D}}{\rm{y}}{\rm{e}}+{\rm{s}}{\rm{u}}{\rm{n}}{\rm{l}}{\rm{i}}{\rm{g}}{\rm{h}}{\rm{t}}\to {{\rm{D}}{\rm{y}}{\rm{e}}}^{\ast }$$

3 $${{\rm{Dye}}}^{\ast }+{\rm{Iso}}-{\rm{AgNPs}}\to {\rm{Dye}}{\cdot }^{+}+{\rm{Iso}}-{\rm{AgNPs}}({{\rm{e}}}_{{\rm{CB}}}^{-})$$

4 $$({{\rm{O}}}_{2})+{\rm{Iso}}-{\rm{AgNPs}}({{\rm{e}}}_{{\rm{CB}}}^{-})\to {\rm{Iso}}-{\rm{AgNPs}}+({{\rm{O}}}_{2}{\cdot }^{-})$$

5 $$({{\rm{O}}}_{2}{\cdot }^{-})+{{\rm{H}}}^{+}\to {{\rm{HO}}}_{2}\cdot $$

6 $$2{{\rm{HO}}}_{2}\cdot \to {{\rm{O}}}_{2}+{{\rm{H}}}_{2}{{\rm{O}}}_{2}$$

7 $${{\rm{H}}}_{2}{{\rm{O}}}_{2}+({{\rm{O}}}_{2}{\cdot }^{-})\to {{\rm{HO}}}^{-}+{\rm{HO}}\cdot +{{\rm{O}}}_{2}$$

8 $${{\rm{e}}}^{-}+{{\rm{H}}}_{2}{{\rm{O}}}_{2}\to {\rm{HO}}\cdot +{{\rm{HO}}}^{-}$$

9 $$({{\rm{H}}}_{2}{\rm{O}})+{\rm{Iso}}-{\rm{AgNPs}}({{\rm{h}}}_{{\rm{VB}}}^{+})\to {\rm{Iso}}-{\rm{AgNPs}}+({\rm{HO}}\cdot )+({{\rm{H}}}^{+})$$

10 $$({{\rm{HO}}}^{-})+{\rm{Iso}}-{\rm{AgNPs}}({{\rm{h}}}_{{\rm{VB}}}^{+})\to {\rm{Iso}}-{\rm{AgNPs}}+({\rm{HO}}\cdot )$$

11 $$\begin{array}{c}{\rm{Dye}}+{\rm{Iso}}-{\rm{AgNPs}}({{\rm{O}}}_{2}{\cdot }^{-},{\rm{HO}}\cdot ,{{\rm{h}}}_{{\rm{VB}}}^{+}\,{\rm{or}}\,\cdot {\rm{OOH}})\to \to \to {{\rm{H}}}_{2}{\rm{O}}\\ \,+{{\rm{CO}}}_{2}+{\rm{byproducts}}+{\rm{Free}}\,{\rm{Iso}}-{\rm{AgNPs}}\,{\rm{for}}\,{\rm{reuse}}\end{array}$$

**Figure 9:**
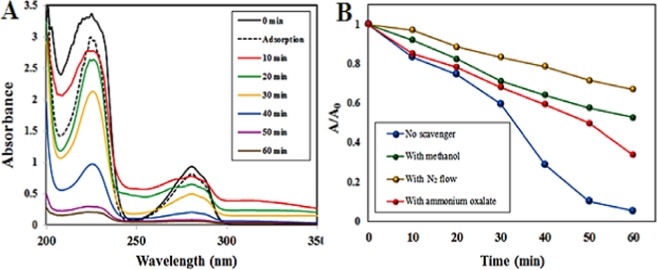
(**A**) UV-vis spectra of the aqueous solution of 40 ppm 4-CP in the presence of Iso-AgNPs photocatalyst. (**B**) The impact of different radical scavengers on the photocatalytic degradation of 4-CP by Iso-AgNPs under natural sunlight.

For approving that the fading of dye is photo-degradation, not a dye sensitization, carcinogenic 4-chlorophenol (4-CP) as a colorless organic pollutant was selected as a target to measure the photocatalytic activity. The evolution of UV-vis spectra of carcinogenic 4-CP under natural sunlight in the presence of the Iso-AgNPs is presented in Fig. 9A; the intensities of peaks at 225 nm and 280 nm characteristic of 4-CP, was decreased in a function of difference irradiation times. Although many aspects regarding the detailed degradation mechanisms of the 4-CP remain unidentified and open to further scrutiny, some primary degradation pathway includes a series of hydroxylation, dehydrogenation, dechlorination and aromatic ring cleavage steps^56–59^. According to the literature, decomposition of 4-CP to H_2_O and CO_2_ molecules as the final products often is a complex process occurring through three primary intermediates: benzoquinone (BQ), hydroquinone (HQ), and 4-chlorocatechol (4-CC)^60,61^. The characteristic absorption peaks of these compounds emerge as intermediates at similar wavelengths as those of 4-CP, i.e. at 246 nm for BQ, 221 nm and 290 nm for HQ, 221 and 284 nm for 4-CC^62^. As can be seen, a gradual decrease of both absorption peaks of 4-CP during photocatalytic procedure may be an indication of decomposition of 4-CP. On the other hand, the lack of the absorption peak characteristic of BQ intermediates at 246 nm in the spectra observed in Fig. 9A suggests that reaction pathway in the presence of Iso-AgNPs is via 4-CC intermediate^62^.

As shown in Fig. 9A, prior to the photocatalytic tests, the adsorption of 4-CP was evaluated. However, the dark physical adsorption of the 4-CP after 60 min onto the nano-photocatalysts was negligible (point line, Fig. 9A) while in photocatalytic process, the decrease of the absorption band of 4-CP was quick. This notable decrease indicated that the removal was also mainly originated from the photocatalytic degradation, not from physical adsorption.

To well understand the presence and role of active species in the photocatalytic degradation route of the organic compounds, the scavenger quenching experiments for 4-CP photo-degradation was selected and carried out at 40 ppm 4-CP concentration for 60 min. Various scavengers used in the present study were ammonium oxalate as an H^+^ scavenger, methanol as an HO• scavenger, and in deoxygenated solutions to conquer the formation of O_2_^−^• by oxygen reduction.

As indicated in Fig. 9B, by addition of oxalate, a slight inhibitory effect was found where around 66% of 4-CP was degraded under natural sunlight. Conversely, deoxygenation of the 4-CP solution significantly decreased the 4-CP molecule photocatalytic degradation rate, where only 34% of 4-CP is removed. Similarly, the existence of methanol leads to an impressive decrease in 4-CP degradation, where about 48% of 4-CP is degraded under natural sunlight. This suggests that the superoxide radicals and then hydroxyl radicals play an important role and acts as main reactive species in the pollutant photo-decomposition. The 4-CP decomposition efficiency was 95.0% without using any scavenger.

### Electrochemical properties of Iso-AgNPs

**Figure 10:**
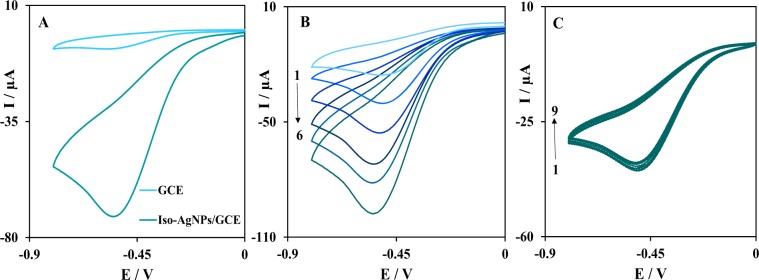
(**A**) Cyclic voltammograms of GCE and Iso-AgNPs/GCE in N_2_-saturated 0.1 M PBS (pH 7.4) containing 1 mM H_2_O_2_ at a scan rate of 100 mV/s, (**B**) Cyclic voltammograms of Iso-AgNPs/GCE in the presence of various concentrations of H_2_O_2_ (peak No.1 to 6 correspondent to conc. 0.15, 0.3, 0.6, 1, 1.3 and 1.6 mM H_2_O_2_, respectively), (**C**) Repetitive cyclic voltammograms (peak No. 1 to 9) of Iso-AgNPs/GCE in the presence of H_2_O_2_.

The electrocatalytic activity of the Iso-AgNPs as a binder‐free catalyst for H_2_O_2_ reduction was investigated by recording cyclic voltammograms in presence of the H_2_O_2_. Figure 10A shows the electrochemical reduction of 1.0 mM H_2_O_2_ at Iso-AgNPs/GCE (Glassy Carbon Electrode) in N_2_-saturated 0.1 M PBS (pH 7.4) at the scan rate of 100 mV/s. As can be seen, at the unmodified GCE, there is no obvious current for the reduction of H_2_O_2_. However, the presence of Iso-AgNPs on the electrode surface had a great improvement on the electrocatalytic reduction of H_2_O_2_, which indicated that the Iso-AgNPs facilitate the electron transfer and increased active surface area. Furthermore, the binder-free Iso-AgNPs can further improve electrochemical performance and electron kinetics of H_2_O_2_ reduction. In the following, to investigate the sensitivity of Iso-AgNPs on the reduction of H_2_O_2_, the cyclic voltammograms were obtained at a different concentration of H_2_O_2_ in N_2_-saturated 0.1 M PBS (pH 7.4), (Fig. 10B). As expected, the peak current increased with the increasing concentration of H_2_O_2_, indicating the catalytic activity of Iso-AgNPs to the reduction of H_2_O_2_. Also, the sensing stability of binder‐free Iso-AgNPs was examined by successive cyclic voltammograms in the presence of H_2_O_2_. As shown in Fig. 10C, after 9 cycles, the reduction peak currents only varied ~6%, indicating that Iso-AgNPs has good stability on the electrode surface for electrochemical reduction of H_2_O_2_. Therefore, the as-prepared Iso-AgNPs could be used as an electrocatalyst without any binder and shows the acceptable stability on the electrode surface.

**Figure 11:**
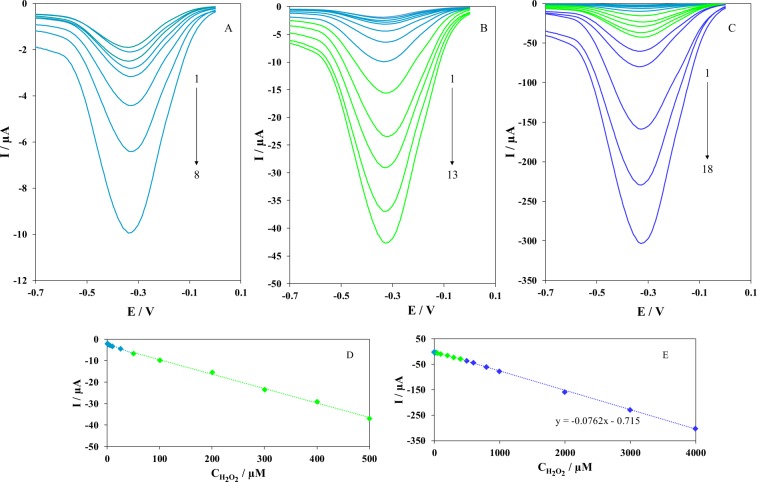
DPVs of Iso-AgNPs/GCE in N_2_-saturated 0.1 M PBS (pH 7.4) containing different concentrations of H_2_O_2_; (**A**) Peak No. 1 to 8 correspondent to 0.1 to 50 μM of H_2_O_2_, (**B**) Peak No. 1 to 13 correspondent to 0.1–500 μM, (**C**) Peak No. 1 to 18 correspondent to 0.1–4000 μM, (**D**,**E**) Plot of the peak currents as a function of H_2_O_2_ concentration, peaks 1–13 and 1–18 respectively.

Differential pulse voltammetry (DPV) was used for the analytical performance of the electrochemical sensor based on Iso-AgNPs toward the reduction of H_2_O_2_. Figure 11 shows the DPV response of Iso-AgNPs/GCE obtained at different H_2_O_2_ concentration in N_2_-saturated 0.1 M PBS (pH 7.4). As expected, the response of Iso-AgNPs/GCE corresponding to the H_2_O_2_ increased with increasing the concentration of H_2_O_2_. The calibration curves of the H_2_O_2_ sensor showed that there was a linear relationship between the electrochemical responses and H_2_O_2_ concentrations from 0.1 µM to 4 mM. Based on the calibration curves in Fig. 11D,E, the plot of electrochemical responses vs. H_2_O_2_ concentration formed a line that had an equation of I (μA) = 0.0762 CH_2_O_2_ (μM) + 0.715. Furthermore, the limit of detection (LOD) was estimated as 0.036 μM from the calibration plot with a signal-to-noise ratio of 3. The results of the proposed electrochemical sensor based on Iso-AgNPs are comparable with other modified electrode for H_2_O_2_ sensing and are displayed in Table 1^63–72^. However, our electrochemical sensor is characterized by a binder‐free, simple design and easy preparation in contrast with other sensors previously described.

**Table 1:** A comparison with other sensors for the detection of H_2_O_2_.

Material of sensor	Linear range	LOD	Reference
Prussian Blue	0.01 μM – 10 mM	0.01 μM	^63^
Cobalt nitride nanowire array on Ti	0.1 μM − 2.5 mM	50 nM	^64^
Pt NPs-carbon quantum dots on GO	1 μM − 900 μM	0.1 μM	^65^
Gr supported intermetallic PtPb NPs	2 nM − 2.5 mM	2 nM	^66^
Silver nanowire	0.1 mM − 3.1 mM	29.2 μM	^67^
GO decorated with silver NPs	0.1 mM – 5 mM	1.099 μM	^68^
Ag@C@Ag trilaminar core-shell	70 μM – 10 mM	23 μM	^69^
Iridium (III) complex	8.0 pM − 2.0 nM	3.2 pM	^70^
Fe_3_O_4_ nanoparticles	4.18 μM − 0.8 mM	1.4 μM	^71^
Co metal-organic framework	5 μM − 9.0 mM	3.76 μM	^72^
Iso-AgNPs	0.1 µM − 4 mM	0.036 μM	This work

## Conclusion

Isoimperatorin, isolated from *Prangos ferulacea* roots, was used successfully to synthesize silver nanoparticles as a simple and cost-effective, precise and eco-friendly methodology. Preparation of Iso-AgNPs was confirmed by UV–Vis spectroscopy and characterization was conducted using XRD, FTIR, SEM, EDX, Raman spectroscopy, TEM and HRTEM. The Iso-AgNPs reveal photo-catalytic performance under sunlight irradiation and can be used for the improvement of the environment through the purification of water. Also, Iso-AgNPs shows the excellent electrocatalytic activity toward H_2_O_2_ as an appropriate option in many biological systems without extra binder and conductive additives. Altogether, the synthesized nanoparticles demonstrate a broad range of catalytic applications.

## Materials and Methods

### Materials

Silver nitrate salt (AgNO_3_), MB, NF, EB, 4-CP, ammonium oxalate, methanol, heptane, ethyl acetate, and silica gel were bought from Merck, Germany. Deuterochloroform (CDCl_3_) was supplied from Sigma–Aldrich, USA. Other reagents, solid materials, Silica gel used in gravity column chromatography and solvents were bought from Merck (Germany).

## Experimental

### Apparatus

A Young Lin apparatus equipped with PDA detector (YL 9160) and a binary pump (YL 9111 S) together with VERTISEP (Reversed-phase, RP18 250 × 30 mm) and Eurospher II (Normal phase, Si 250 × 20 mm) columns with flow rate of 10 mL/min have been used for high-performance liquid chromatography (HPLC) analysis.

The NMR spectrum of Isoimperatorin was recorded on a BRUCKER (500 MHz) instrument, using CDCl_3_ as a solvent. MS analysis was performed on an AGILENT 6410 Triple Quadrupole mass spectrometer (AGILENT Technologies, Palo Alto, CA, USA)^73^ coupled with an AGILENT Mass Hunter Workstation B.01.03. TLC plates (Silica gel 60 GF254 precoated plates, Merck) were analyzed by UV observation at 254 and 366 nm, and by spraying with cerium sulfate/ molybdate^74^.

The synthesis of Iso-AgNPs was confirmed by UV–vis spectrometer (SHIMADZU, Lambda UV mini-1240 instrument) in the absorption wavelength range of 200 to 700 nm. The FTIR of isoimperatorin and Iso-AgNPs was performed by IR-Prestige-21 (SHIMADZU Spectrometer, Kyoto, Japan); KBr Pellets of samples were used to measure transmittance percentage. The spectrum was recorded at a resolution of 4 cm^−1^. The XRD spectrum of the dried nanoparticles was recorded by APD 2000-Italian Structures X-ray generator; to carry out XRD, the samples were coated on the XRD grid, and a voltage of 40 kV with a current of 30 mA using Cu K^−1^ radiation was applied. The SEM and EDX (FESEM-TSCAN, Czech), were performed for the analysis of surface topographies and chemical composition of Iso-AgNPs. Raman spectroscopy was performed by NICOLET-910, USA. The morphological analysis of NPs was evaluated by TEM microscopy (ZEISS – EM10C, Germany) and HRTEM microscopy (FEI – TEC9G20, USA) at an accelerating voltage of 100 kV and 200 kV, respectively. The aqueous suspension of Iso-AgNPs was loaded on the carbon-coated copper grid. After evaporation of solvent at room temperature for one-hour, clear microscopic views were observed in the different range of magnifications. The light intensity in the place of the sample was gotten from Kermanshah weather bureau. Electrochemical studies were conducted with an AUTOLAB PGSTAT101 potentiostat/galvanostat (ECO CHEMIE, Netherlands) with standard three-electrode system, using GCE or a modified GCE as working electrode, a platinum wire as a counter electrode and Ag/AgCl/KCl as a reference electrode.

### Isolation of isoimperatorin

Isoimperatorin was isolated from *Prangos ferulacea* roots as described previously^75^. In brief, a MeOH-soluble (in −20 °C) part of acetone extract of roots was fractionated using heptane ethyl acetate (7:3) as the eluant and silica gel as the stationary phase. The final purification was performed by normal phase preparative HPLC.

Isoimperatorin; EI-MS m/z 270 [M]^+^, 255 [M-CH3], 227 [M-(CH3)2CH] ^+^.H^1^NMR (CDCl_3_, 500 MHz): 8.19 d (1 H, J: 9.6, H-4), 7.62 d (1 H, *J*: 2.3, H-2′), 7.19 s (1 H, H-8), 6.99 d (1 H, *J*: 2.3, H-3′), 6.30 d (1 H, *J*: 9.6, H-3), 5.75 t (1 H, *J*: 6.8, H-2′′), 4.95 d (2 H, *J*: 6.8, H-1′′), 1.84 s (3 H, CH3–5′′), 1.73 s (3 H, CH3–6′′).

### Synthesis of AgNPs with isoimperatorin

The isoimperatorin solution (2 mmol/L) was obtained by dissolving isoimperatorin in 2 mL ethanol. Then, the ethanol solution was added dropwise to 6 mL deionized water and incubated for 10 min at room temperature. Then, the isoimperatorin solution was quickly added to 12 mL aqueous solution of AgNO_3_ (1 mmol/L). Finally, the reaction was placed under sunlight (The light intensity was 25.5 MJ/m^2^) to change the color from pale yellow to reddish-brown which indicates the formation of Iso-AgNPs.

### Photocatalytic properties of Iso-AgNPs

Using the four kinds of hazardous pollutant involve three dyes model (MB, NF, and ER) and carcinogenic 4-CP as a non-dye pollutant, the photocatalytic activity of Iso-AgNPs was demonstrated under sunlight irradiation by a slightly modified method of Roy *et al*.^76^. Primarily, the dye and non-dye solutions were prepared at a concentration of 10 mg/L and 40 mg/L in DI water respectively. Afterward, 5 mg of Iso-AgNPs was added to 25 mL of each dye solution (at first, powder of Iso-AgNPs was sonicated in 5 mL DI water for 15 min and then added to 20 mL pollutant solution). These colloidal suspensions were then placed under natural sunlight irradiation with constant stirring. The average temperature of the ambiance during the test was around 14 °C (geographical coordinates: 34.3277°N and 47.0778°E) and the light intensity was 7.7 MJ/m^2^ for MB, NF, and ER. In the case of 4-CP, the average temperature was around 21 °C and the light intensity was 9.9 MJ/m^2^. At regular intervals time (every 15 min for dye model and every 10 min for 4-CP), 2 mL suspension was taken from the colloidal mixture and centrifuged at 5,000 rpm for15 min to obtain clean supernatant of the tested pollutant. Finally, the absorbance maxima of dye models at different time intervals (0, 15, 30, 45, 60 min) was monitored using UV–visible spectrophotometer at a different wavelength from 200 to 800 nm for evaluation of dye degradation. The absorbance maxima of 4-CP were monitored at 0, 10, 20, 30, 40, 50 and 60 min time intervals.

### Free radical trapping experiments

The scavenger quenching was investigated by adding scavengers consisting 0.5 mM methanol (a quencher of •OH), nitrogen (a quencher of •O_2_^−^, 0.3 L/min), and 0.5 mM ammonium oxalate (a quencher of h^+^) to evaluate the effect of each radical on the photo-degradation process of the pollutants.

### Electrochemical measurement procedure

Iso-AgNPs modified GCE was constructed by the drop-casting method. Before modification, the working electrode was polished with alumina powder on a mirror-like surface. A volume of 5 µL of the Iso-AgNPs aqueous solution was applied directly on a cleaned GCE surface and drying at room temperature to obtain Iso-AgNPs/GCE. After modification, cyclic voltammetry and differential pulse voltammetry was used to characterize the electrocatalytic activity and stability of the Iso-AgNPs toward reduction of H_2_O_2_ in 0.1 M PBS (pH 7.4). The PBS buffer was purged with ultrapure nitrogen for 10 min to remove dissolved oxygen before the measurement.

### Statistical analysis

One-way Analysis of Variance (ANOVA) and Tukey-Kramer post-test was applied for the determination of differences between groups at p <0.05 level using SPSS statistical package (SPSS, Version 16 for Windows, SPSS Inc., Chicago, USA).
